# Early Warning Signs of Autism: A Systematic Review of Nonverbal Behavioral Markers and Early Developmental Red Flags in Children Under 36 Months

**DOI:** 10.7759/cureus.105485

**Published:** 2026-03-19

**Authors:** Hanan Raed Mushtaha Ahmad, Qabas Hasan Alqudah, Hajer Elmutazbellah Elshikh Elkhalifa, Alaa Shannan Merghani, Ramez Riad Abusbaih, Ahmed Noman Ghaleb, Osman Osama Osman Mohamed Elhassan, Asim Ahmed, Mawadah Jibreel

**Affiliations:** 1 Pediatrics, Mutah University, School of Medicine and Surgery, Zarqa, JOR; 2 Pediatrics, Jordanian Royal Medical Services, Amman, JOR; 3 Pediatrics, National University - Sudan, College of Medicine and Surgery, Khartoum, SAU; 4 General Practice, National University - Sudan, College of Medicine and Surgery, Khartoum, ARE; 5 Faculty of Medicine, Ain Shams University, Cairo, EGY; 6 Faculty of Medicine, Sulaiman Al-Rajhi University, Al Bukayriyah, SAU; 7 General Practice, Faculty of Medicine and Surgery, Omdurman Islamic University, Omdurman, QAT; 8 Medicine and Surgery, University of Gezira, Wad Madani, SDN; 9 Dentistry, Gadarif Teaching Hospital, Gadarif, SDN

**Keywords:** autism spectrum disorder, early identification, infancy, nonverbal behavioral markers, systematic review

## Abstract

Early identification of autism spectrum disorder (ASD) remains a priority because clinical diagnosis often occurs after the earliest stages of development, and early behavioral signs can be subtle and variable across children. This systematic review summarized evidence on early nonverbal behavioral markers measured before 36 months that are associated with later-ASD diagnosis or classification. A PRISMA-aligned search was conducted. Eligible studies assessed nonverbal behavioral markers before age three and reported ASD outcomes at a later follow-up. Data were extracted on study design, population, marker domains, assessment methods, and predictive performance when reported. Risk of bias was assessed using tools matched to the study type. Findings were synthesized narratively because studies differed in cohorts, tasks, and outcome definitions. Across the included studies, early ASD-related signals most often appeared as differences in developmental change over time rather than as fixed abnormalities present from birth. Evidence was most consistent for differences in social visual engagement and attention flexibility during the first year of life, with more apparent differences from late infancy into the second year in attention disengagement, repetitive and sensory-linked behaviors, and motor and postural development. Predictive performance for single markers was generally limited. At the same time, combinations across domains and multimodal approaches appeared more useful, although they still require external validation and more transparent reporting before use in practice. Overall, the evidence supports longitudinal surveillance that combines multiple early behavioral domains to improve early risk identification.

## Introduction and background

Autism spectrum disorder (ASD) is a neurodevelopmental condition defined by persistent differences in social communication and social interaction, alongside restricted and repetitive patterns of behavior, interests, or activities. Symptoms begin in early development and can affect daily functioning. ASD is etiologically and phenotypically heterogeneous, which contributes to wide variation in the timing and pattern of early signs across children [1,2].

For example, some children may exhibit different social attention trajectories, with one child steadily improving in social attentiveness, while another shows initial strength followed by a decline as they age. Surveillance data show a substantial population burden and reinforce that early identification remains a public health priority, as many children are not recognized at the earliest ages when developmental pathways may be most responsive to support [3].

Clinical practice guidelines emphasize developmental surveillance and timely assessment when ASD is suspected. Guidance from the American Academy of Pediatrics highlights the need for structured evaluation of children with concerning social communication development and timely connection of families to appropriate services [4]. Updated recommendations continue to stress systematic identification, comprehensive evaluation, and ongoing management in pediatric care [5]. Standardized screening tools are commonly used, including the Modified Checklist for Autism in Toddlers, Revised with Follow-up (MCHAT-RF), which improves screening performance in toddler-aged children [6]. However, most screening tools are designed for toddlerhood and rely on caregiver report and clear behavioral features, which may be subtle or not yet stable during the first year of life. For this reason, there is continued interest in earlier indicators that can be measured before the usual age of clinical diagnosis [4-6]. In this review, nonverbal behavioral markers refer to observable early behaviors that do not depend on spoken language, such as patterns of eye contact, shifts in attention, motor behaviors, and sensory responses.

Research recommendations have emphasized identifying developmentally sensitive markers that can be detected in infancy and linked to later-ASD outcomes. Consensus statements further highlight the need to define candidate early markers, clarify measurement approaches, and distinguish early risk signals from features that may emerge later as part of atypical development [7,8]. A major driver of this work is the use of enriched risk cohorts, especially familial high-risk cohorts, such as infant siblings of children with ASD, because familial recurrence is higher than that in the general population and allows prospective tracking from early infancy. Great collaborative efforts have quantified recurrence risk and refined estimates across cohorts, supporting longitudinal designs that examine early developmental change before diagnosis [9,10].

Early ASD-related signals are often conceptualized as developmental differences that emerge over time rather than fixed deficits present from birth. Evidence from early indicators suggests that detectability depends on the measured domain, the developmental timing of assessment, and the measurement method [11,12]. In particular, early indicators have been described across nonverbal domains, including social attention, sensory motor behaviors, and early regulation. Still, the specific pattern and timing can vary among children and across settings [11,12]. Conceptual and clinical discussions of early diagnosis also emphasize that symptoms may be difficult to identify in very young children reliably and that early risk signals should be interpreted within a broader developmental context [13]. Together, these findings support the view that early nonverbal behaviors may be measurable indicators of later-ASD outcomes, although their timing and predictive value can vary by method and population [11-13].

The importance of early identification is further supported by evidence that earlier intervention can improve outcomes in young children with ASD. Randomized trial evidence for intensive early developmental intervention, including the Early Start Denver Model, supports the premise that earlier identification can be consequential not only for diagnosis but also for developmental trajectories and family support [14]. Despite this, the literature on early nonverbal behavioral markers remains fragmented across many tasks, heterogeneous cohorts, and variable outcome definitions, limiting clear conclusions about which markers appear earliest and which provide the strongest evidence for prediction.

Accordingly, this systematic review synthesizes evidence on early nonverbal behavioral markers measured before 36 months that are associated with later-ASD diagnosis or classification. The primary objective is to identify and synthesize early nonverbal behavioral markers associated with later-ASD diagnosis or classification when measured before 36 months. Secondary objectives are to map the earliest age at which each marker can be detected across prespecified windows of 0 to 6 months, 6 to 12 months, 12 to 18 months, and 18 to 36 months; compare evidence across assessment approaches, including direct observation, video or free play coding, parent report, and standardized tools; summarize predictive validity metrics and key effect estimates when reported, including sensitivity, specificity, positive predictive value, negative predictive value, and area under the curve; describe how study population type such as high-risk siblings versus general population and study design influence marker detection and performance; and identify which combinations of marker domains, such as motor development, attention disengagement, or the ability to shift attention away from one stimulus to another, and repetitive behaviors show the most substantial evidence for early identification.

## Review

Methods

Review Design and Objectives

This systematic review was designed to synthesize early nonverbal behavioral markers associated with later ASD diagnosis in children under 36 months. The objectives were to identify the earliest detectable markers; map detection timing across age windows of 0 to 6 months, 6 to 12 months, 12 to 18 months, and 18 to 36 months; compare evidence across assessment approaches; summarize predictive validity metrics when reported; examine whether population type and study design influence detection and performance; and identify combinations of marker domains with the most substantial evidence for early identification. This review followed the PRISMA 2020 guidance [15]. This review was not prospectively registered.

Eligibility Criteria Using PICO (Population, Indicator or Intervention, Comparison, and Outcome)

Eligibility criteria were defined using the PICO framework, including population, exposure or index markers, comparator groups, outcomes, and eligible study designs (Table 1).

**Table 1:** Eligibility criteria using PICO framework ASD: Autism spectrum disorder; AOSI: Autism Observation Scale for Infants; PICO: Population, indicator or intervention, comparison, and outcome.

Element	Criteria
Population (P)	Human infants and toddlers assessed before 36 months of age. This included high familial-risk cohorts, for example, infant siblings of children with ASD as well as general population or low-risk comparison groups.
Exposure or index (I)	Early nonverbal behavioral markers measured in infancy or toddlerhood. Domains included social attention and gaze behavior, attention disengagement and visual regulation, motor and postural development, repetitive or sensory-linked behaviors, and standardized observational instruments, for example, AOSI.
Comparator (C)	Children who did not meet ASD outcome criteria, low-risk infants, typically developing controls, or other clinical comparison groups, for example, developmental delay, as defined in each included study.
Outcomes (O)	The primary outcome was later-ASD diagnosis or ASD classification using the study-defined diagnostic standards. When reported, predictive performance metrics and effect estimates were extracted, including sensitivity, specificity, positive predictive value, negative predictive value, and area under the curve.
Study designs	Prospective longitudinal cohort designs were prioritized. Comparative or cross-sectional designs in later toddlerhood were included when they directly evaluated early nonverbal markers relevant to ASD classification or prediction.

Information Sources and Search Strategy

A systematic literature search was developed to capture studies assessing early nonverbal behavioral markers of ASD risk in infants and toddlers. We searched PubMed, the Cochrane Library, and Web of Science. Searches were conducted from database inception to 15 December 2025. The search strategy combined controlled vocabulary, when available, and free-text keywords covering ASD, the developmental period, and early marker domains. Reference lists of included studies were also checked to identify additional eligible papers. The full database-specific search strategies are provided in Appendix 1. The overall approach followed standard systematic review methods (Table 2) [16].

**Table 2:** Information sources and search strategy ASD: Autism spectrum disorder; AOSI: Autism Observation Scale for Infants; PRISMA: Preferred Reporting Items for Systematic Reviews and Meta-Analyses.

Component	Description
Databases searched	PubMed, Cochrane Library, Web of Science
Date range	Database inception to 15 December 2025
Search concepts	ASD terms, developmental period terms, and early marker domain terms
Keywords and terms used	ASD: autism, autism spectrum disorder, ASD. Developmental period: infant, infancy, toddler. Marker domains: eye tracking, gaze, social attention, visual disengagement, attention shifting, sticky attention, motor development, head lag, posture, repetitive behavior, stereotyped behavior, object exploration, AOSI, Autism Observation Scale for Infants.
Other sources	Reference lists of included studies were checked for additional eligible papers.
Methods guidance	PRISMA 2020 reporting guidance [15] and standard systematic review methods [16]

Study Selection

Records identified through the search were deduplicated before screening. Titles and abstracts were screened against the eligibility criteria, and full-text reviews were conducted for potentially relevant articles. Two reviewers performed study selection. Disagreements were resolved by discussion and consensus. The final set included in this manuscript spans references [17-36], which are dominated by prospective longitudinal cohort evidence, with a smaller number of comparative or cross-sectional studies.

Data Extraction

Data were extracted using a standardized extraction framework aligned with the review objectives. Extracted variables included study design, population and risk group, sample size, infant age at marker assessment, marker domain and operational definition, assessment method, timing of ASD outcome ascertainment, and the main findings. Predictive validity metrics and modeling outputs were extracted when reported. When these details were not reported clearly in the source article, they were recorded as not reported and not imputed. Two reviewers performed data extraction. Disagreements were resolved by discussion and consensus.

Risk-of-Bias Assessment

Risk of bias was assessed using tools matched to study type. Prognostic factor and longitudinal association studies were appraised using the QUIPS (Quality in Prognosis Studies) tool. Diagnostic accuracy studies were assessed using QUADAS-2 (Quality Assessment of Diagnostic Accuracy Studies). Measurement property studies were evaluated using the COSMIN (COnsensus-based Standards for the selection of health Measurement Instruments) checklist. Multivariable prediction model studies were assessed with PROBAST (Prediction model Risk-Of-Bias ASsessment Tool). Two reviewers performed the risk-of-bias assessment, and disagreements were resolved through discussion and consensus. As several judgments were necessarily derived from extracted or abstract-level information, some domains were rated as unclear when reporting was insufficient to confirm key safeguards, such as confounding control, missing-data handling, or validation procedures.

Data Synthesis

Given heterogeneity in populations, tasks, marker operationalization, and outcome definitions, findings were synthesized qualitatively rather than pooled quantitatively. Meta-analysis was not conducted because this heterogeneity precluded meaningful quantitative pooling across studies. Predictive metrics and effect estimates were summarized descriptively when reported. The synthesis was structured by prespecified age windows of 0 to 6 months, 6 to 12 months, 12 to 18 months, and 18 to 36 months and by marker domains, including social attention, attention disengagement and visual regulation, motor development, repetitive or sensory-linked behavior, and multimodal prediction approaches.

Results

PRISMA Flow and Study Selection

**Figure 1:**
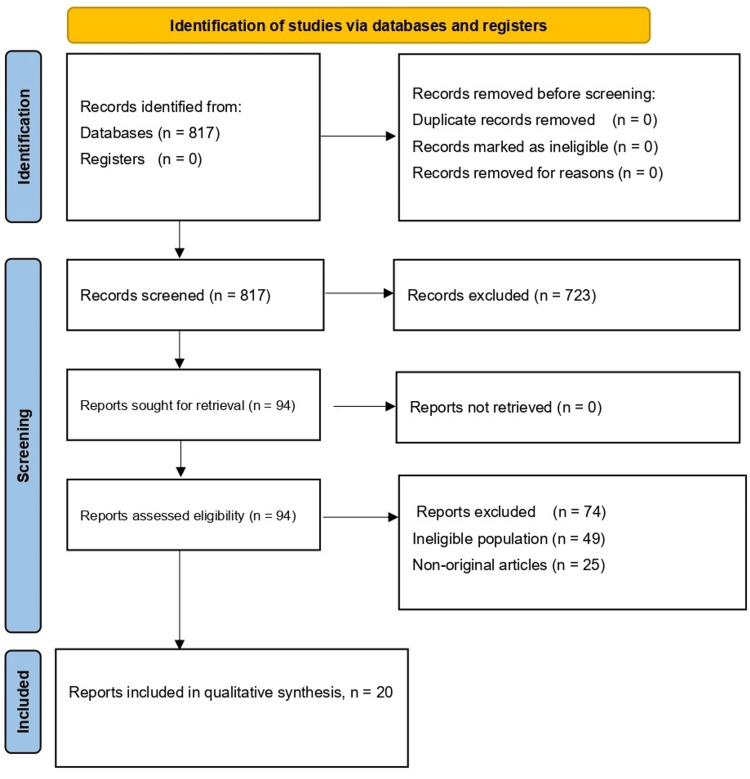
PRISMA 2020 flow diagram of study selection (n = 20 included) The diagram summarizes record identification from databases (n = 817), screening (records excluded, n = 723), full-text eligibility assessment (reports assessed, n = 94; excluded, n = 74), and final inclusion in qualitative synthesis (n = 20). PRISMA: Preferred Reporting Items for Systematic Reviews and Meta-Analyses.

The database search identified 817 records, none of which were identified from registers. No duplicate records were removed before screening, and no records were removed for other reasons. All 817 records were screened by title and abstract, and 723 were excluded for failing to meet eligibility criteria. Complete text reports were sought for 94 records, and all 94 were retrieved. A total of 94 full-text reports were assessed for eligibility. Of these, 74 reports were excluded, including ineligible population (n = 49) and non-original articles (n = 25). Finally, 20 studies were included in the qualitative synthesis. The study selection process is shown in the PRISMA 2020 flow diagram (Figure 1).

Study Characteristics

Across references [17-36], the evidence was dominated by prospective longitudinal cohort designs. Most studies enrolled infant siblings at familial high risk, meaning an older sibling had ASD, alongside low-risk comparison infants. A smaller number of studies used comparative or cross-sectional designs in later toddlerhood [18-20,22-27,29-36].

Assessments spanned early infancy through 42 months. ASD outcomes were typically determined between 24 and 36 months, and one study extended follow-up to seven years [18,20,23,24,26,29,31,32,34,36]. Marker assessment was mainly based on eye-tracking paradigms, video-coded observational tasks, and standardized observational instruments, such as Autism Observation Scale for Infants (AOSI). These approaches covered domains of social attention, attention disengagement and visual regulation, motor development, repetitive behaviors, and multimodal prediction models [17-20,22-25,28-33,35,36]. Study characteristics and extracted markers are summarized in Table 3.

**Table 3:** Included studies and extracted early nonverbal markers associated with later-ASD diagnosis ASD: Autism spectrum disorder; HR: High risk, meaning familial risk due to an older sibling with ASD; LR: Low risk; AOSI: Autism Observation Scale for Infants; MSEL: Mullen Scales of Early Learning; ADOS: Autism Diagnostic Observation Schedule; CSBS: Communication and Symbolic Behavior Scales; RSMS: Repetitive and Stereotyped Movement Scales; PDMS: Peabody Developmental Motor Scales; DD: Developmental delay; NDD: Neurodevelopmental disorder; PPV: Positive predictive value. Source: Refs. [17-36].

Study	Design	Population	Sample size	Age at assessment	Marker domain	Marker(s) extracted	Assessment method	ASD outcome timing	Key findings
Jones and Klin (2013) [17]	Prospective longitudinal	Infants later ASD vs non-ASD	Not reported	2–6 months	Social attention	Decline in eye fixation to eyes across 2–6 months (not absent at start)	Eye-looking/eye fixation tracking (instrument not specified)	Later-ASD diagnosis (age not reported)	Later-ASD infants showed initially typical eye-looking followed by a decline from two to six months; non-ASD infants did not show this trajectory.
Elsabbagh et al. (2013) [18]	Prospective cohort	Familial risk vs no familial risk	104	7 and 14 months	Attention disengagement	Longer disengagement latency at 14 months; atypical 7→14 month change	Visual disengagement task (central-to-peripheral orienting)	Diagnosis at 36 months	No robust association at seven months; by 14 months, slower disengagement in later-ASD subset; atypical developmental change was also seen in some non-ASD developmental concerns.
Chawarska et al. (2013) [19]	Prospective	High-risk vs low-risk infants	67 HR; 50 LR	6 months	Social attention	Reduced attention to social scenes and face monitoring; no compensatory object attention	Eye-tracking task	Clinical outcome in the third year	At six months, later-ASD infants showed diminished spontaneous attention to people/activities and reduced face monitoring.
Ozonoff et al. (2010) [20]	Prospective longitudinal	High-risk vs low-risk typical	25 later ASD; 25 LR typical	6, 12, 18, 24, and 36 months	Social communication	Declining trajectories of gaze to faces, social smiles, and directed vocalizations	Video-coded behaviors + examiner ratings	Outcome by 36 months	Groups similar at six months; later-ASD group showed declining trajectories with differences by 12 months; skill loss was not well captured by parent-reported regression.
Bedford et al. (2014) [21]	Prospective high-risk study	Low-risk controls and high-risk infants later diagnosed with ASD	Not reported	13 months	Social + nonsocial attention	Gaze following and attentional disengagement independently predictive (additive model)	Gaze following + disengagement tasks (abstract-level description)	Diagnosis at three years	Social (gaze following) and nonsocial (disengagement) attention measures each independently predicted ASD outcome; best fit described by an additive model.
Ibanez et al. (2008) [22]	Cross-sectional sibling comparison	ASD-sibs vs siblings of typically developing children	17 vs 17	6 months	Visual attention control	Fewer gaze shifts to/from parent’s face; longer gazes away; no difference in face-looking duration	Face-to-face/Still-face protocol; gaze coding	Not reported	ASD-sibs showed intact time looking at faces but greater interest in non-face stimuli (less shifting + longer away looks).
Sacrey et al. (2013) [23]	Prospective longitudinal cohort	High-risk siblings + non-sibling controls	Not reported	6–36 months (repeated)	Visual attention regulation	Prolonged disengagement latency (“sticky attention”) from 12–24 months	Video-recorded play with graspable toys; latency/duration indices	Diagnostic assessment at 36 months	Sticky attention emerged by 12 months and persisted through 24 months in later-ASD infants.
Ozonoff et al. (2008) [24]	Prospective cohort	Followed prospectively; later ASD vs comparison groups	66 total; ASD = 9	12 months	Repetitive behavior/object exploration	Spinning, rotating, unusual visual exploration	Structured object exploration; blinded coding	Outcome at 36 months	The later-ASD group showed more spinning/rotating/unusual visual exploration; early repetitive behaviors related to later cognitive/symptom status.
Kaur et al. (2015) [25]	Longitudinal observational	At-risk vs typically developing	16 vs 16	6–15 months (repeated)	Object exploration	Grasping, mouthing, looking, purposeful dropping (context-dependent)	Video-recorded task (three objects) with coded behaviors	Follow-up at 18 months and after the second birthday	Multiple age- and object-dependent differences in sensorimotor exploration; some at-risk infants later had delays/ASD diagnosis.
Flanagan et al. (2012) [26]	Prospective + replication	High-risk siblings + low-risk comparison	Sample 1: 40 HR; Sample 2: 20 HR + 21 LR	6 months	Motor/postural control	Head lag during pull-to-sit	Clinical pull-to-sit assessment	Diagnosis at 36 months	Head lag at six months associated with later-ASD classification; more frequent in high-risk than low-risk.
Zwaigenbaum et al. (2005) [27]	Longitudinal high-risk cohort	High-risk siblings + low-risk comparison	HR = 150 (subset followed to 24 months emphasized)	6–24 months	Multidomain phenotype	Social attention, orienting to name, imitation, affect, sensory-oriented behaviors, temperament, language; prolonged disengagement	Observational scale + computerized orienting task + standardized measures	Outcome assessed through 24-month follow-up	By ~12 months, later-ASD infants differed across multiple social attention and sensory markers, with prolonged disengagement and emerging temperament/language differences.
Bryson et al. (2008) [28]	Tool development/reliability	High-risk infants	Not reported	6, 12, 18 months	Observational tool	AOSI total score + endorsed items reliability	AOSI	Not an outcome study	AOSI total score and endorsed items showed good–excellent inter-rater reliability across 6/12/18 months; item-level reliability was more modest at six months.
Zwaigenbaum et al. (2021) [29]	Prospective cohort	High-risk siblings vs low-risk	501 HR; 180 LR	6–18 months	Multidomain early ASD features	AOSI total score; ROC cutoffs at 12 and 18 months	AOSI	Diagnosis at age 3	AOSI differentiated later-ASD beginning at 12 months; reported sensitivity/specificity values indicated limitations for stand-alone screening.
Pierce et al. (2011) [30]	Comparative eye-tracking	ASD vs developmental delay vs typical	110 (ASD = 37; DD = 22; TD = 51)	14–42 months	Visual preference	Fixation preference for dynamic geometric patterns	Split-screen movie + eye-tracking	Cross-sectional classification	ASD toddlers fixated more on geometric images; high fixation threshold yielded a high PPV in this sample.
Landa and Garrett-Mayer (2006) [31]	Prospective cohort	High-risk siblings + low-risk infants	87	6, 14, 24 months	Global development	MSEL domain trajectories	MSEL + ADOS + clinical judgment	Outcome grouping at 24 months	No group differences at six months; ASD group lower by 14 months and lower across domains by 24 months, with a slower developmental trajectory.
Elison et al. (2014) [32]	Cohort comparison	LR vs HR-negative vs HR-ASD	53, 75, 30	12 months	Repetitive behaviors	Motor stereotypies; repetitive object manipulation; composite score	Observational coding from CSBS using RSMS	ASD assessed at 24 months	HR-ASD showed more motor stereotypies than other groups; repetitive object manipulation was higher in HR groups; graded composite across LR < HR-negative < HR-ASD.
Gliga et al. (2015) [33]	Prospective high-risk longitudinal	High familial-risk siblings	Not reported	9 months (predictor); later outcomes	Perception/visual search	Enhanced visual search	Eye-tracking; letter-target visual search paradigm	Symptoms at 15 months and 2 years	Enhanced visual search at nine months predicted higher autism symptom levels later.
LeBarton and Landa (2019) [34]	Prospective cohort	High- and low-familial-risk infants	140	6 months (predictor)	Motor development	Early motor vulnerability (grasping, visual–motor integration, and postural skills)	PDMS subscales	ASD at 24–36 months; language at 30/36 months	Six-month motor skills predicted later-ASD status and later expressive language.
Caruso et al. (2020) [35]	Prospective trajectory study	High-risk siblings + low-risk infants; grouped by outcome	LR = 53; HR = 50	10 days to 24 weeks	Motor trajectories	Quantitative kinematic features	Motion-tracking (MOVIDEA)	NDD outcome classification	Early motor parameter trajectories differed in high-risk infants later classified with NDD outcomes.
Bedford et al. (2017) [36]	Prospective with mid-childhood follow-up	High-risk siblings + low-risk controls	Not reported	7 and 14 months; follow-up at 7 years	Neurocognitive + observational	Early neurocognitive markers + AOSI	Neurocognitive measure(s) + disengagement task + AOSI	ASD at 7 years	Neurocognitive markers distinguished later ASD; combining neurocognitive markers with AOSI improved prediction over AOSI alone.

Qualitative Synthesis by Age Window and Marker Domain

Findings from the first six months suggested that early signals may be interpreted as developmental changes over time rather than fixed deficits. In a prospective longitudinal study, infants later diagnosed with ASD showed typical early eye-looking followed by a decline in eye fixation from two to six months. This pattern was not observed in infants who did not develop ASD [17]. At six months, reduced spontaneous monitoring of social scenes and reduced face monitoring were also observed in infants later diagnosed with ASD in a high-risk versus low-risk design [19]. Subtler differences in visual attention control were reported in infant siblings at familial risk. They showed fewer gaze shifts to or from a parent’s face and longer periods of looking away, despite similar overall time spent looking at the face compared with sibling controls [22]. Early motor and postural vulnerability was also reported, with head lag at six months associated with later-ASD classification in a high-risk cohort with replication and low-risk comparison sampling [26].

Between 6 and 12 months, several studies reported limited early group separation, with more apparent differences emerging as development progressed. In a prospective repeated-measures design from 6 to 36 months, social communication behaviors, including gaze to faces, social smiles, and directed vocalizations, were similar at six months and then showed declining trajectories in the later-ASD group, with differences evident by 12 months [20]. Attention regulation markers also showed timing effects. Visual disengagement at seven months was not strongly associated with later outcome, whereas at 14 months, longer disengagement latencies were observed in the subgroup later diagnosed with ASD, alongside atypical developmental change from 7 to 14 months [18]. Perceptual markers were also identified in this period, with enhanced visual search at nine months predicting higher autism symptom levels later in toddlerhood [33].

From 12 to 18 months, evidence was more consistent for differences in attention control and repetitive behaviors. A longitudinal play-based paradigm reported prolonged disengagement latency, often called sticky attention, emerging by 12 months and persisting through 24 months in infants later diagnosed with ASD [23]. In a high-risk study that tested a multifactor framework, gaze following and attention disengagement, assessed at 13 months, each independently predicted ASD outcome at three years, supporting an additive risk model rather than a single-deficit pathway [21]. Repetitive behaviors and atypical object exploration were also detectable around 12 months. Atypical object exploration behaviors, such as spinning, rotating, and unusual visual exploration, were associated with later ASD in a prospective sample [24]. Motor stereotypies at 12 months were also elevated in infants later diagnosed with ASD compared with high-risk infants without ASD and low-risk infants, with a graded pattern across risk groups [32]. Broader developmental divergence was also described, with minimal differences at six months but more apparent differences by 14 and 24 months across multiple developmental domains [31].

From 18 to 36 months, studies included standardized observational tools and specific eye-tracking paradigms. The AOSI showed acceptable inter-rater reliability in high-risk samples [28]. In a large prospective cohort, AOSI total scores differentiated high-risk infants later diagnosed with ASD beginning at 12 months. Reported sensitivity increased by 18 months; however, these values were insufficient for stand-alone screening [29]. In a comparative toddler sample aged 14-42 months, fixation preference for dynamic geometric patterns was higher among children with ASD, and a high fixation threshold yielded a high positive predictive value in that sample [30]. Motor development findings were not limited to early infancy. Motor skill at six months predicted later expressive language and ASD diagnosis at 24-36 months in a prospective cohort [34]. Objective movement tracking trajectories in the first 24 weeks also differentiated high-risk infants later classified with neurodevelopmental disorders. This may support early surveillance even when ASD was not the only endpoint [35].

Beyond toddlerhood, one longitudinal study reported that infant neurocognitive markers, including early neural responses to gaze shifts and later disengagement latency, distinguished children with ASD diagnoses at seven years and improved prediction when combined with an observational marker, the AOSI [36].

Reported Predictive Performance

Given heterogeneity in marker tasks, measurement scales, and outcome reporting across studies, effect estimates were not sufficiently similar to support quantitative pooling. Predictive metrics reported in the included studies are summarized in Table 4.

**Table 4:** QUIPS risk of bias for prognostic marker studies Judgments are based on extracted table summaries and abstract-level information; therefore, some domains may be rated as unclear due to incomplete reporting. QUIPS: Quality In Prognosis Studies; RoB: Risk of bias; ASD: Autism spectrum disorder; NDD: Neurodevelopmental disorder. Source: Refs. [17-20,22-27,31-35].

Study	Design	QUIPS overall RoB	Main reason
Jones et al. (2013) [17]	Prospective longitudinal	Moderate	Attrition/missingness, confounding control, and analysis details not fully available from extraction.
Elsabbagh et al. (2013) [18]	Prospective cohort	Moderate	Confounding/adjustment and missing-data handling unclear; enriched familial-risk sampling limits generalizability.
Chawarska et al. (2013) [19]	Prospective	Moderate	Marker measurement clear, but selection/generalizability and analytic control reporting limited in extraction.
Ozonoff et al. (2010) [20]	Prospective longitudinal	Moderate	Trajectory approach strong; missing-data handling and confounder adjustment not clearly reported here.
Ibanez et al. (2008) [22]	Cross-sectional sibling comparison	High	Small sample and no ASD outcome timing/diagnosis reporting → high risk in participation/outcome/analysis domains.
Sacrey et al. (2013) [23]	Prospective longitudinal	Moderate	Repeated measures help, but attrition/missingness and confounding control not fully captured in extraction.
Ozonoff et al. (2008) [24]	Prospective cohort	High	Very small later-ASD group (ASD=9) increases instability and analysis/reporting bias risk.
Kaur et al. (2015) [25]	Longitudinal observational	High	Small sample and mixed follow-up outcomes; limited clarity on confounding and analytic plan.
Flanagan et al. (2012) [26]	Prospective + replication	Moderate	Replication strengthens credibility; remaining concerns relate to blinding/missingness/confounding reporting.
Zwaigenbaum et al. (2005) [27]	Longitudinal high-risk cohort	Moderate	Multiple markers increase multiple-testing risk; confounding and attrition reporting unclear in extraction.
Landa and Garrett-Mayer (2006) [31]	Prospective cohort	Moderate	Strong cohort design; confounding control and missingness handling not clearly available here.
Elison et al. (2014) [32]	Cohort comparison	Moderate	Observational coding appropriate; concerns mainly from unclear confounding adjustment and missingness reporting.
Gliga et al. (2015) [33]	Prospective high-risk longitudinal	Moderate	Predictive association reported; validation/transportability and confounding control unclear from extraction.
LeBarton and Landa (2019) [34]	Prospective cohort	Moderate	Prognostic signal plausible; adjustment set and missingness/attrition handling not fully specified here.
Caruso et al. (2020) [35]	Prospective trajectories	High	Outcome classified as NDD (not ASD-specific) and modeling/feature selection may inflate optimism without clear validation.

Risk of Bias

Risk of bias was assessed using methods matched to the study's purpose and design. Studies evaluating early markers as prognostic factors for later-ASD outcomes were appraised using the QUIPS instrument. Studies that reported cutoff or threshold performance in a diagnostic accuracy framework were appraised using QUADAS 2. The study focused on the measurement reliability of the AOSI, which was appraised using a COSMIN-style reliability framework. Studies combining multiple predictors to improve prediction were appraised using PROBAST for prediction modeling. Two reviewers performed the risk-of-bias assessment. Disagreements were resolved by discussion and consensus. As the available information came from extracted table summaries and abstract-level summaries, several tool-specific items, including attrition, missing-data handling, blinding, prespecified confounding control, and validation strategy, could not be confirmed for all studies. This increased the number of judgments rated as moderate or high risk of bias.

Under QUIPS, most prognostic marker studies were judged to have a moderate risk of bias. The most common reasons were incomplete reporting of attrition, poor handling of missing data, unclear control for confounding, and limited transparency about analysis choices. Higher risk was mainly observed in studies with minimal later-ASD subgroups, cross-sectional risk group comparisons without later-ASD outcome confirmation, or outcome definitions that were not specific to ASD, such as broader neurodevelopmental disorder endpoints. These judgments are summarized in Table 4.

**Figure 2:**
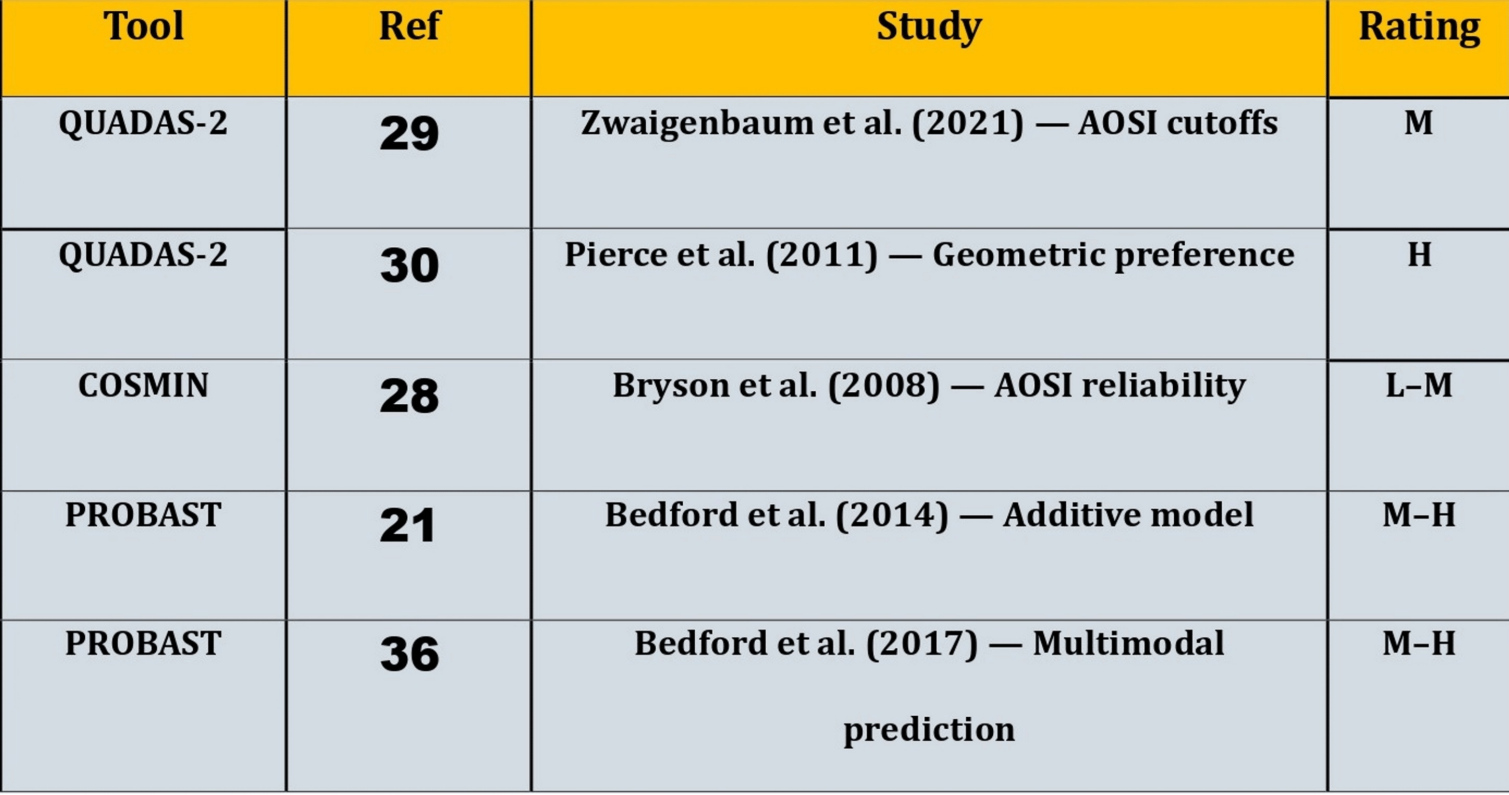
Risk-of-bias overview for non-QUIPS studies by tool (n = 5) L: Low risk; M: Moderate concerns; H: High risk; M–H: Moderate–high concerns; L–M: Low–moderate; QUIPS: Quality in Prognosis Studies; QUADAS: Quality Assessment of Diagnostic Accuracy Studies; COSMIN: COnsensus-based Standards for the selection of health Measurement Instruments; PROBAST: Prediction model Risk-Of-Bias ASsessment Tool; AOSI: Autism Observation Scale for Infants. Image credit: The authors of the current study.

The smaller set of non-QUIPS studies showed a different pattern. The threshold-based diagnostic accuracy study was judged to be at high risk of bias due to spectrum concerns and the threshold being set and tested within the same sample. The large prospective cohort that reported AOSI cutoffs had fewer selection concerns, but uncertainty remained about transportability and external validation. The AOSI reliability study was judged as low to moderate risk for measurement properties. The multivariable prediction studies were considered to have moderate to high risk because external validation and optimism bias adjustment could not be confirmed from the extracted information. These judgments are summarized in Figure 2.

Discussion

Principal Findings

This systematic review synthesizes early nonverbal behavioral markers associated with later-ASD diagnosis in children assessed under 36 months. Across the included evidence, the overall pattern is most consistent with an emerging divergence model, in which group differences become clearer over time rather than presenting as fixed abnormalities from birth [17,18,20,31,36]. Early changes are observed across multiple systems, particularly social visual engagement, attention regulation, motor development, and repetitive or sensory-linked behaviors, but no single marker is sufficient for reliable prediction across settings [21,26,32,34,36].

Interpretation Across Developmental Domains

Jones and Klin (2013) described one of the clearest examples of developmental change by showing that eye-looking can appear typical early and then decline between two and six months in infants later diagnosed with ASD [17]. This trajectory-based pattern contrasts with approaches that focus on single-time-point differences. A plausible interpretation is that an early, typical profile, followed by a decline, reflects the timing of neurodevelopmental specialization and shifting social demands in early infancy [17]. The implication is that longitudinal measurement may be more sensitive than one-off screening in very early infancy.

Elsabbagh et al. (2013) examined attention disengagement. They found that associations with later ASD were less clear at seven months but clearer by 14 months, with longer disengagement latencies in infants later diagnosed with ASD [18]. In the same developmental window, Chawarska et al. (2013) reported reduced spontaneous attention to social scenes at six months in infants later diagnosed with ASD [19]. One possible explanation for why attention differences appear earlier in social-orienting tasks than in disengagement tasks is that task demands and construct definitions differ across the two tasks. Social scene viewing captures spontaneous attention allocation, whereas disengagement tasks target a specific control process that may mature later [18]. The implication is that early detection may depend on matching the task to the developmental stage and the mechanism being tested.

Ozonoff et al. (2010) provided strong longitudinal evidence that many social communication differences are not apparent at six months but emerge over time, with declining trajectories in gaze to faces, social smiles, and directed vocalizations that become clearer by 12 months [20]. This supports the idea that some early markers reflect differences in the rate of change rather than the early absence of skills. A further interpretation is that repeated assessments and trajectory modeling may reveal divergence that a single observation can miss [20]. The implication is that surveillance systems should prioritize repeated measurement rather than relying solely on a single-pass screening event.

Bedford et al. (2014) strengthened the multidomain interpretation by demonstrating additive effects of social attention and nonsocial attention measures in infancy, suggesting that multiple pathways can contribute to later-ASD outcome [21]. This contrasts with models that assume a single dominant early deficit. While measuring more signals can mechanically improve prediction, additive models are also consistent with ASD heterogeneity and may reduce reliance on any single unstable marker [21]. The implication is that early risk frameworks should combine domains rather than search for a single decisive behavioral sign.

Ibanez et al. (2008) highlighted that early differences may appear in the structure of gaze behavior rather than in the total face-looking time, with fewer gaze shifts to and from a parent’s face and longer looks away in infant siblings of children with ASD [22]. Sacrey et al. (2013) then reported attention patterns during play consistent with prolonged disengagement, often framed as sticky attention, becoming more apparent across the first and second years in infants later diagnosed with ASD [23]. Together, these studies suggest that attention-related risk may be visible in both interactive and play-based contexts, but the most informative metric may change with age [22,23]. The implication is that marker choice should be developmentally staged and context specific.

Ozonoff et al. (2008) reported that atypical object exploration at 12 months, including spinning, rotating, and unusual visual inspection, was associated with later ASD [24]. Kaur et al. (2015) described atypical trajectories in object exploration across 6 to 15 months, including persistent mouthing and less purposeful dropping, and illustrated challenges related to mixed outcomes and small samples in at-risk cohorts [25]. These findings complement social attention markers by highlighting sensory motor exploration as a relevant early domain. A key interpretive issue is specificity. Atypical exploration can occur across multiple neurodevelopmental pathways, making the choice of comparators and the definition of outcomes central [25]. The implication is that repetitive and sensory-linked behaviors may support risk stratification, but they should be interpreted within a broader developmental profile.

Flanagan et al. (2012) reported that head lag during pull-to-sit at six months was associated with later-ASD classification and was more frequent in high-risk infants than low-risk controls [26]. This offers a clinically accessible marker compared with eye-tracking measures, but it also raises concerns about specificity because postural control differences can occur in other developmental conditions. Zwaigenbaum et al. (2005) broadened the early phenotype description by reporting behavioral manifestations across the first year, including sensory-oriented behaviors and differences in orienting, supporting the view that early risk is multidomain rather than isolated [27]. The implication is that motor and regulatory markers may contribute to early identification, but they should be combined with other domains to reduce false positives [26,27].

Bryson et al. (2008) focused on measurement reliability by reporting the development and inter-rater reliability of the AOSI [28]. Reliability is necessary for clinical translation, but reliability alone does not guarantee predictive validity. Zwaigenbaum et al. (2021) evaluated AOSI symptom measurement from 6 to 18 months in a large high-risk cohort. They reported that AOSI scores differentiated infants later diagnosed with ASD from around 12 months, while performance was not sufficient for stand-alone screening [29]. A plausible explanation is that base-rate effects, overlapping early behaviors among high-risk infants, and the limited transportability of thresholds constrain stand-alone accuracy [29]. The implication is that observational tools may be most useful within staged surveillance and referral pathways rather than in isolation through universal early screening.

Pierce et al. (2011) reported that preference for dynamic geometric patterns over social images showed intense discrimination in their sample, with a high positive predictive value at a specific fixation threshold [30]. This differs from gradual divergence markers by suggesting a potentially strong classifier within a particular task. However, threshold selection and evaluation in the same sample can inflate performance and may not generalize across settings. Without external validation and transparent reporting of the threshold-derivation process, performance should be interpreted cautiously [30]. The implication is that such measures may be promising for enriching research on high-risk cohorts but require replication across diverse populations before clinical adoption.

Landa and Garrett-Mayer (2006) reported minimal group differences at six months on broad developmental measures, with more apparent divergence between 14 and 24 months across multiple domains [31]. This supports the view that global developmental scales may be less sensitive in early infancy than targeted microlevel measures. Elison et al. (2014) reported elevated repetitive behavior at 12 months in infants later classified with ASD, with patterns that distinguished outcome groups in some cohorts [32]. Together, these findings reinforce the notion that detectability depends on the outcome domain and measurement granularity [31,32]. These findings suggest that early identification strategies should not rely solely on broad developmental tests but should integrate targeted measures that capture specific processes.

Gliga et al. (2015) reported enhanced visual search in infancy predicting emerging autism symptoms, indicating that early markers may include attentional strengths or biases, not only delays [33]. LeBarton and Landa (2019) reported that infant motor skills at six months predicted later-ASD diagnosis and later expressive language outcomes, supporting a developmental cascade interpretation in which early motor differences may shape later learning opportunities [34]. The implication is that early markers across perceptual and motor systems may converge on broader developmental allocation patterns that influence later social communication. However, direction and specificity may vary by cohort [33,34].

Caruso et al. (2020) used motion-tracking to examine early motor development. They reported that early movement trajectories predicted later clinical outcomes in high-risk siblings, with outcomes sometimes framed more broadly as neurodevelopmental disorders rather than ASD diagnosis alone [35]. This highlights a key issue. Broader outcome classification may increase sensitivity at the expense of ASD specificity, and this trade-off should be explicit when interpreting clinical usefulness [35]. The implication is that objective sensing may support early developmental surveillance. Still, translation requires clarity on which outcomes are being predicted and how well approaches differentiate ASD from other developmental pathways [35].

Finally, Bedford et al. (2017) extended follow-up into mid-childhood and reported that combining early neurocognitive markers with observational measures improved predictive accuracy compared with observational measures alone [36]. This supports multimethod integration and is consistent with ASD risk spanning multiple systems. Improved performance likely reflects reduced dependence on any single measurement context. However, confirmation in independent cohorts remains essential [36]. The implication is that future early identification research should prioritize multidomain, multimethod models with prespecified analysis plans, robust handling of missing data, and external validation across diverse populations and service contexts [36].

Clinical and Research Implications

Integrated across studies, the evidence supports developmentally staged surveillance that combines multiple early behavioral domains and prioritizes repeated measurements over time [17,18,20,21,31,36]. For practice, the findings support careful monitoring of attention regulation, social visual engagement, motor and postural development, and emerging repetitive or sensory-linked behaviors, particularly in high-risk infants, while avoiding overinterpretation of any single signal [22-27,32,34]. For research, the next step is not simply to identify more candidate markers but to standardize definitions, improve reporting of attrition and missing-data handling, reduce optimism in predictive modeling through appropriate validation, and test transportability across cohorts with differing risk status, demographic characteristics, and clinical pathways [30,36].

Limitations and Future Directions

An additional limitation is that the search was restricted to the prespecified databases and did not include a dedicated trial registry search. Although this approach captured the main published literature, relevant studies indexed in other databases or registered outside these sources may have been missed.

Overall, the included studies suggest that early ASD-related differences are most consistently expressed as changes in developmental trajectories, with the earliest and strongest signals involving social visual engagement and attention regulation, followed by more apparent differentiation in motor and repetitive or sensory-linked behaviors as infants approach the second year [17-20,23,26,32,34]. As single markers show limited predictive value, the evidence supports longitudinal surveillance that combines multiple domains and emphasizes external validation of multimodal approaches before clinical adoption [21,29,30,36].

## Conclusions

This review indicates that ASD-related behavioral differences may be detectable before the typical age of diagnosis. Still, the most informative signals appear to reflect developmental change over time rather than isolated single-time point deficits. Across studies, convergent domains included attentional flexibility, social visual engagement, early motor and postural development, and emerging repetitive or sensory-linked behaviors, with more apparent differentiation most often reported from late infancy into the second year.

These findings support a staged approach to early surveillance that integrates multiple domains and repeated assessment over time, rather than relying on any single marker. Future research should prioritize standardized marker definitions and outcomes, transparent reporting of missing-data handling and analytic decisions, and broader external validation of prediction models across diverse cohorts to improve transportability and clinical utility.

## Appendices

Appendix 1 

**Table 5:** Full database specific search strategies ASD: Autism spectrum disorder; AOSI: Autism Observation Scale for Infants; TS: Topic search; ti,ab,kw: Title, abstract, and keywords; tiab: Title and abstract; MeSH: Medical Subject Headings.

Database	Search date	Full search string	Limits or filters
PubMed	December 15, 2025	((Autism Spectrum Disorder[Mesh] OR autism[tiab] OR autism spectrum disorder[tiab] OR ASD[tiab]) AND (infant[tiab] OR infants[tiab] OR infancy[tiab] OR toddler[tiab] OR toddlers[tiab] OR early childhood[tiab] OR newborn[tiab] OR neonatal[tiab] OR under 36 months[tiab] OR first year[tiab] OR second year[tiab]) AND (eye tracking[tiab] OR gaze[tiab] OR social attention[tiab] OR visual disengagement[tiab] OR attention disengagement[tiab] OR attention shifting[tiab] OR sticky attention[tiab] OR motor development[tiab] OR head lag[tiab] OR posture[tiab] OR repetitive behavior[tiab] OR repetitive behaviors[tiab] OR stereotyped behavior[tiab] OR object exploration[tiab] OR AOSI[tiab] OR Autism Observation Scale for Infants[tiab] OR nonverbal behavioral marker*[tiab] OR early marker*[tiab]))	No database filters applied at the search stage
Cochrane Library	December 15, 2025	((autism spectrum disorder OR autism OR ASD):ti,ab,kw) AND ((infant OR infants OR infancy OR toddler OR toddlers OR early childhood OR newborn OR neonatal OR under 36 months OR first year OR second year):ti,ab,kw) AND ((eye tracking OR gaze OR social attention OR visual disengagement OR attention disengagement OR attention shifting OR sticky attention OR motor development OR head lag OR posture OR repetitive behavior OR repetitive behaviors OR stereotyped behavior OR object exploration OR AOSI OR Autism Observation Scale for Infants OR nonverbal behavioral marker* OR early marker*):ti,ab,kw)	No database filters applied at the search stage
Web of Science	December 15, 2025	TS=((autism OR autism spectrum disorder OR ASD) AND (infant OR infants OR infancy OR toddler OR toddlers OR early childhood OR newborn OR neonatal OR under 36 months OR first year OR second year) AND (eye tracking OR gaze OR social attention OR visual disengagement OR attention disengagement OR attention shifting OR sticky attention OR motor development OR head lag OR posture OR repetitive behavior OR repetitive behaviors OR stereotyped behavior OR object exploration OR AOSI OR Autism Observation Scale for Infants OR nonverbal behavioral marker* OR early marker*))	No database filters applied at the search stage
